# Land use dominate the evolution of ecosystem services in the Huaihe River Eco-Economic Belt, China

**DOI:** 10.1038/s41598-026-47378-w

**Published:** 2026-04-09

**Authors:** Wenhui li, Bin Yang, Ru Feng, Xinchuan Li, Jiaoyuan Wang, Huaijun Wang

**Affiliations:** 1 School of Geography and Planning, Huaiyin Normal University, Huai’an, 223300 China; 2 School of Computer Science and Technology, Huaiyin Normal University, Huai’an, 223300 China; 3 State Key Laboratory of Hydrology-water Resources and Hydraulic Engineering, Nanjing Hydraulic Research Institute, Nanjing, 210029 China

**Keywords:** Ecosystem services, InVEST model, Land use, Huaihe River Eco-Economic Belt, Ecology, Environmental sciences

## Abstract

The Huaihe River Eco-Economic Belt, located in the north-south transition zone of China, is a pivotal component of the national economic system. Quantitative analysis of the evolution and driving mechanisms of ecosystem services is essential for achieving regional sustainable development. Using land-use data from 1990, 2000, 2010, and 2020, we employed the InVEST model and the equivalent factor method to simulate variations in five ecosystem services: carbon sequestration, habitat quality, water yield, food‑material supply, and potential soil erosion. Hierarchical partitioning and stepwise regression were applied to reveal the impacts of land‑use proportions on ecosystem services. The results show that (1) carbon sequestration, habitat quality, and food‑material supply declined, while potential soil erosion and water yield increased; (2) synergistic relationships were observed among the five ecosystem services, with strong synergies between habitat quality and carbon sequestration, and between water yield and food‑material supply; (3) forest, urban and rural, and dry land proportions were the most significant factors affecting carbon sequestration and habitat quality. Forest proportion explained more than 40% of the variation, while urban and rural, dry land accounted for 30% and 17%, respectively. Land‑use proportions explained about 50% of the variation in potential soil erosion and water yield. For food‑material supply, paddy field proportion explained 37% of the variation, with dry land, urban–rural land, and forest each contributing about 15%. These findings provide valuable insights for land‑use planning and management in the Huaihe Eco‑economic Belt.

## Introduction

Ecosystem services are fundamental benefits and environmental conditions that sustain human survival and development, directly or indirectly provided by ecosystems^1,2^. Since the inception of ecosystem service studies, they have become essential benchmarks for assessing ecological security, ecological red lines, and key ecological functional zones, and are crucial for landscape planning^3–5^. The Millennium Ecosystem Assessment classifies ecosystem services into four categories: (i) provisioning services, which include products obtained from ecosystems; (ii) regulating services, which provide benefits from ecosystem process regulation; (iii) cultural services, which refer to non-material benefits such as spiritual satisfaction, cognitive development, and aesthetic experience; and (iv) supporting services, which are necessary for the production of all other services^6^. Interactions among ecosystem services are complex but can be categorized into trade-offs and synergies^7–9^. Trade-offs occur when the increase of one service reduces another, while synergies arise when multiple services increase simultaneously^10^. Assessing ecosystem service values, analyzing the relationships among them, and finally achieving a balance between ecological conservation and economic development are crucial for regional development and environmental protection.

In recent years, the evaluation of ecosystem services receives extensive attention from scholars not only from fields of ecology, geography but also from economy etc.^11,12^. The InVEST model, a set of freely accessible and open-source software tools developed by the Natural Capital Project for modeling and mapping ecosystem services, is the most extensively used model for evaluating ecosystem service globally^13,14^. It includes three ecosystem service evaluation models for land, freshwater, and marine ecosystems^15^. Recent studies have demonstrated that InVEST can precisely quantify diverse ecosystem service functions in different regions, thus providing an effective decision-making tool for balancing different ecosystem service functions and spatial changes^16^. For example, InVEST has been applied in assessing water yield in data scarce region of northwest China^17^, conducting habitat assessment in South Africa^18^, evaluating carbon sequestration in a reserve in India^19^, and calculating potential soil erosion in a small basin with semi-arid climate in north China^20^, yielding remarkable outcomes.

In addition to quantitatively assessing various ecosystem services, exploring the relationships between ecosystem services has also attracted attention^21–24^. Zheng et al.^21^ conducted a meta-analysis to identify the most common trade-offs among ecosystem services, and found that provisioning services and regulating services/biodiversity was the most common trade-offs. In urban-rural complexes, trade-offs occurred mainly between land-dependent services (such as crop production and water supply), synergies occurred mainly between land-independent services (such as manufactured products and students of higher education)^23^. Howe et al.^7^ revealed that trade-offs are recorded almost three times as often as synergies after identifying 1324 relevant reports. They also pointed out that considering why trade-offs occurred is more likely to create mutually beneficial situations than planning for a mutually beneficial from the outset. Hence, it is essential to comprehend the trade-offs and synergies among different ecosystem services to facilitate effective management and conservation of ecosystems. The analysis, as mentioned above, suggests that, although the InVEST model has made considerable progress in the evaluation ecosystem service values, specific issues remain unaddressed. Firstly, most studies focus on particular regions or cities, with limited research conducted within the broader economic belt, which is a region implementing system coordination planning among multiple administrative districts. Secondly, though the bulk of the literature centers around trade-off relationships among different ecosystem services, no consistent relationships were found. In addition, land use change is the primary driver of changes in ecosystem services; however, further research is needed to determine which specific type of land use change is more significant in certain ecosystem service.

In this study, we focused on the Huaihe River Ecological Economic Belt, China (hereafter referred to as Huaihe River Eco-Economic Belt), an area with dense population, developed economy, and transit climate. Using land use data from four periods (1990, 2000, 2010, and 2020), we integrate the InVEST model with the hierarchical partitioning method to: (1) conduct a systematic evaluation of ecosystem service values, (2) assess the potential trade-offs among the ecosystem services; and (3) identify the dominant land use type controlling different ecosystem services. The results of this study can provide valuable insights for informed land-use planning and ecological protection in the Huaihe River Eco-Economic Belt.

## Materials and methods

### Study area

**Fig. 1:**
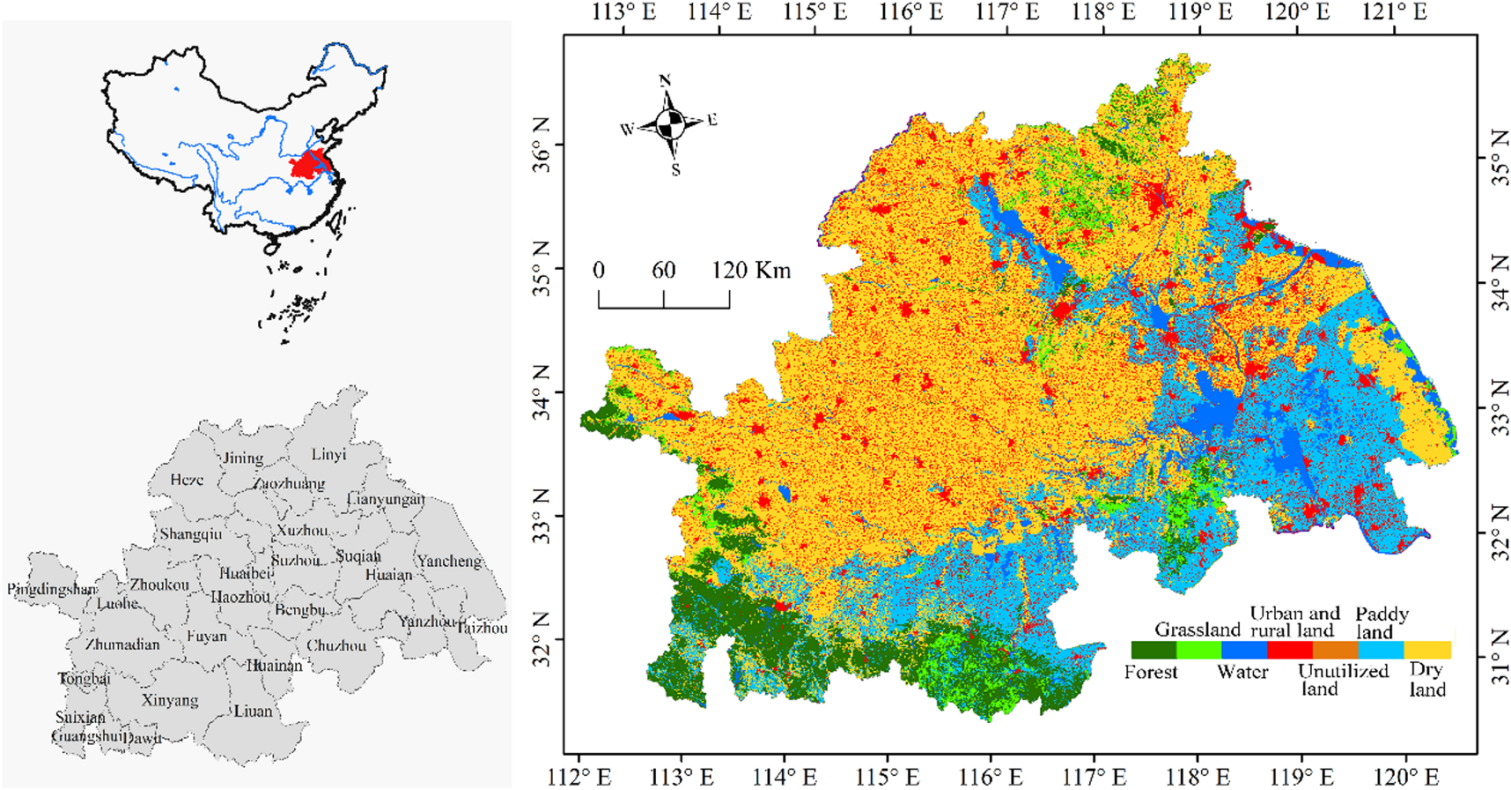
Location of the Huaihe River Eco-Economic Belt and distribution of land-use types in 2020.

The Huaihe River Eco-Economic Belt was elevated to a national strategy on 18 October 2018, following the State Council’s approval of the Development Plan of the Huaihe Ecological Economic Belt. It covers an area of approximately 243,000 km², including Suixian, Guangshui, and Dawu counties in Hubei Province; seven prefecture-level cities (e.g., Zhumadian, Pingdingshan) in Henan Province; eight cities (e.g., Huainan, Chuzhou) in Anhui Province; seven cities (e.g., Huaian, Yangzhou) in Jiangsu Province; and four cities (e.g., Linyi, Heze) in Shandong Province (Fig. 1). Geographically, the Huaihe River Eco-Economic Belt lies between 111°55′E–121°20′E and 30°55′N–36°20′N. It locates in the transitional climate zone between northern and southern China (Fig. 1). Mean annual precipitation decreases from south to north, the annual mean precipitation is 992.9 mm, and the mean annual temperature is 13.7 °C. The total water resources of the region amount to 8.54 × 10⁹ m³, with surface water contributing approximately 73%. Despite this, the region is characterized by water scarcity and uneven distribution of water resources. In addition, per capita water resources are only one-quarter of the national average. The Huaihe River Eco-Economic Belt is also marked by high population density and diverse land-use types (Fig. 1), making it one of the most ecologically vulnerable regions in eastern China.

### Data

This study primarily relied on the InVEST model, which incorporates several modules, including water yield, soil erosion, habitat quality, and carbon sequestration. Food supply was calculated using the equivalent factor method. Land-use data at a spatial resolution of 1 km were obtained from the Resource and Environment Science Data Center of the Chinese Academy of Sciences (http://www.resdc.cn/, accessed 20 May 2021). Four time periods were selected: 1990, 2000, 2010, and 2020. Considering the characteristics of the Huaihe River Eco-Economic Belt, land-use types were reclassified into seven categories: forest, grassland, water, urban and rural land, unused land, paddy land, and dry land (Fig. 1). Monthly precipitation (1901–2020) and potential evapotranspiration (1990–2020) at a 1 km resolution were obtained from the Qinghai-Tibet Plateau Database (https://data.tpdc.ac.cn, accessed 22 May 2021)^25,26^.

#### Carbon sequestration

Carbon sequestration was calculated using the following equation:

1 $${{\mathrm{C}}_{{\mathrm{tot}}}}={{\mathrm{C}}_{{\mathrm{above}}}}+{{\mathrm{C}}_{{\mathrm{below}}}}+{{\mathrm{C}}_{{\mathrm{soil}}}}+{{\mathrm{C}}_{{\mathrm{dead}}}}$$

where C_tot_ represents the total carbon stock (t/hm^2^), C_above_ is the aboveground carbon stock, C_below_ represents the belowground carbon stock, C_soil_ is the soil carbon stock, and C_dead_ denotes the carbon stock in dead organic matter. Carbon density data for the Huaihe River Eco-Economic Belt were derived from statistical yearbooks and previous studies^27,28^, which compiled carbon densities for different land-use types across various regions (Table 1).

**Table 1:** Carbon density of different land-use types in the Huaihe River Eco-Economic Belt (t/hm²).

Land-use type	C_above_	C_below_	C_soil_	C_dead_
Forest	67.45	30.32	136.98	3.40
Grassland	2.83	23.52	138.83	2.90
Water	0.14	0	71.77	0
Urban and rural land	0.02	3.20	64.6	0
Unused land	0.02	4.90	66.02	0
Paddy land	18.90	12.50	85.5	2.40
Dry land	17.90	11.50	80	1.40

#### Water yield

Annual water yield was estimated using the Budyko curve combined with mean annual precipitation. The calculation involved subtracting actual evapotranspiration from precipitation for each raster cell to obtain average annual water depth. The annual mean water yield for each pixel was calculated as:

2 $${\mathrm{Y}}({\mathrm{x}})=\left( {1 - \frac{{1+{\mathrm{AET}}({\mathrm{x}})}}{{{\mathrm{P}}({\mathrm{x}})}}} \right) * {\mathrm{P}}({\mathrm{x}})$$

where AET(x) represents the annual actual evapotranspiration of pixel x, and P(x) is the mean annual precipitation of pixel x. Root-restricting layer depth and plant-available water content (PAWC) were obtained from the ISRIC Data Center (https://data.isric.org, accessed 20 May 2021). Maximum rooting depth and crop coefficient (Kc) are listed in Table 2.

#### Potential soil erosion

The potential soil loss was calculated through the application of the Universal Soil Loss Equation (USLE), as follows:

3 $${\rm USLE = R \times K \times LS \times C \times P_{c}}$$

where USLE represents the annual potential soil erosion (t), R represents the rainfall erosivity factor, K is the soil erodibility factor, LS represents the slope length and steepness factor, P_c_ is the soil conservation measure factor, and C represents the vegetation coverage factor. R is a dynamic index factor that quantifies the erosive power of rainfall. Typically, it is calculated as the product of the total kinetic energy of the rainfall event and the maximum 30-minute rainfall intensity. However, the acquisition of required rainfall data at a requisite spatiotemporal resolution is limited. Therefore, this study adopted an empirical formula that integrated both the annual and monthly average precipitation for the calculation of the rainfall erosivity factor as follows^29–31^:

4 $${\mathrm{R}}=1.2157{\sum\limits_{{i=1}}^{{12}} {10} ^{1.5(\lg {\kern 1pt} P_{{\mathrm{i}}}^{2}/P) - 0.0818}}$$

The formula (4) incorporates three variables, where R represents the yearly rainfall erosivity (MJ·mm/MJ·mm·hm^− 2^·h^− 1^), *P* represents the yearly precipitation (mm), and *P*_*i*_ represents the precipitation for the *i*-th month (mm). K was derived from the National Tibetan Plateau Data Center (https://data.tpdc.ac.cn/zh-hans/data/926339e3-2e27-44a2-a829-7623795759fc/, accessed on 20 May 2021), and the dataset is “Soil Erodibility Dataset of Pan-Third Pole 20 countries”. Detailed parameters for each land-use type are listed in Table 2.

**Table 2:** Land-use attributes used for water yield and soil conservation modeling in the Huaihe River Eco-Economic Belt.

Land-use type	C	*P*_c_	Root depth (mm)	Kc
Forest	0.025	1	7000	1.008
Grassland	0.034	1	2600	0.85
Water	0	1	10	0.975
Urban and rural land	0.99	1	1	0.2
Unused land	0.85	1	5000	0.75
Paddy land	0.412	0.2	2000	1.125
Dry land	0.412	0.6	2000	0.954

#### Habitat quality

Habitat quality was assessed using the following equation:

5 $${Q_{xj}}={H_j} \times \left[ {1 - \left( {\frac{{D_{{xj}}^{z}}}{{D_{{xj}}^{z}+{k^z}}}} \right)} \right]$$

where *Q*_*xj*_ is the habitat quality index of grid *x* within land-use type *j*, *H*_*j*_ is the habitat suitability of land-use type *j*, *D*_*xj*_ represents the habitat degradation degree of grid *x* within land-use type *j*, *z* is a normalization constant, and *k* is the half-saturation constant, set to half of the maximum habitat degradation degree^32^. To reflect the local geographical characteristics of the study area, urban and rural land, unused land, paddy land, and dry land were identified as the primary threat sources. Based on local conditions, the maximum impact distance and weight of each stress factor affecting habitat quality were determined (Table 3). Using this approach, we defined the habitat suitability of different land-use types and their relative sensitivity to threat sources (Table 4).

**Table 3:** Impact scope and weights of threat sources affecting habitat quality in the Huaihe River Eco-Economic Belt.

Threat source	Weight	Maximum impact distance (km)
Urban and rural land	1	12
Unused land	1	5
Paddy land	0.5	3
Dry land	0.5	3

**Table 4:** Habitat suitability and relative sensitivity of land-use types to different threat sources.

Land use type	Habitat suitability	Urban and rural land	Unused land	Paddy land	Dry land
Forest	1	0.8	0.7	0.6	0.5
Grassland	1	0.7	0.6	0.5	0.4
Water	1	0.9	0.6	0.5	0.5
Urban and rural land	0	0	0	0	0
Unused land	0	0.5	0.4	0.3	0.3
Paddy land	0	0.7	0.4	0.3	0.3
Dry land	0	0.6	0.5	0.3	0.3

#### Food and raw material supply

Food and raw material supply (hereafter food-material supply) is a primary ecosystem service provided by ecosystems. In this study, we estimated food-material supply using the equivalent factor method developed by Xie et al.^33^. To minimize the impact of regional heterogeneity on ecosystem service value (ESV) assessment, we adjusted the ESV coefficient table at the national scale. The ESV equivalent factor coefficient was derived by considering the relative contribution rates of ecosystem services across different ecosystem types and was set as one-seventh of the market value of the average grain yield per unit area for the corresponding year^34^. We used grain area and yield data, as well as average grain prices for all cities and counties within the Huaihe River Eco-Economic Belt (Jiangsu, Anhui, Henan, Hubei, and Shandong), obtained from the National Compilation of Agricultural Product Cost-Benefit Data for the selected time period. Then we calculated the unit value of the ESV equivalent factor as 1,505.8 Yuan·hm⁻². Using this factor, food-material supply was computed for each land-use type (Table 5).

**Table 5:** Equivalence table of ecosystem service values (ESV) for food-material supply in the Huaihe River Eco-Economic Belt.

Type	Dry land	Paddy land	Forest	Grassland	Water	Urban rural land	Unused land
Food production	1280	2048	380	452	1205	0	0
Raw material production	602	136	873	670	346	0	0
Food raw material production	1882	2183	1254	1122	1551	0	0

### Statistical analysis

We analyzed spatial synergies and trade-offs among pairs of ecosystem services at the city level using Pearson correlation analysis. To quantify land-use effects on ecosystem services, we assembled ecosystem service values and corresponding land-use proportions for 29 cities and counties in 1990, 2000, 2010, and 2020, and performed multiple stepwise regression. We further applied hierarchical partitioning (HP) to quantify the independent contribution of each land-use type. HP effectively deals with the issue of multicollinearity, and is widely used in ecology and geography^35,36^. HP was implemented using the “hier.part” R package^37^. All statistical analyses were conducted in R 3.5.2.

## Results

### Spatial and temporal changes in ecosystem services

**Fig. 2:**
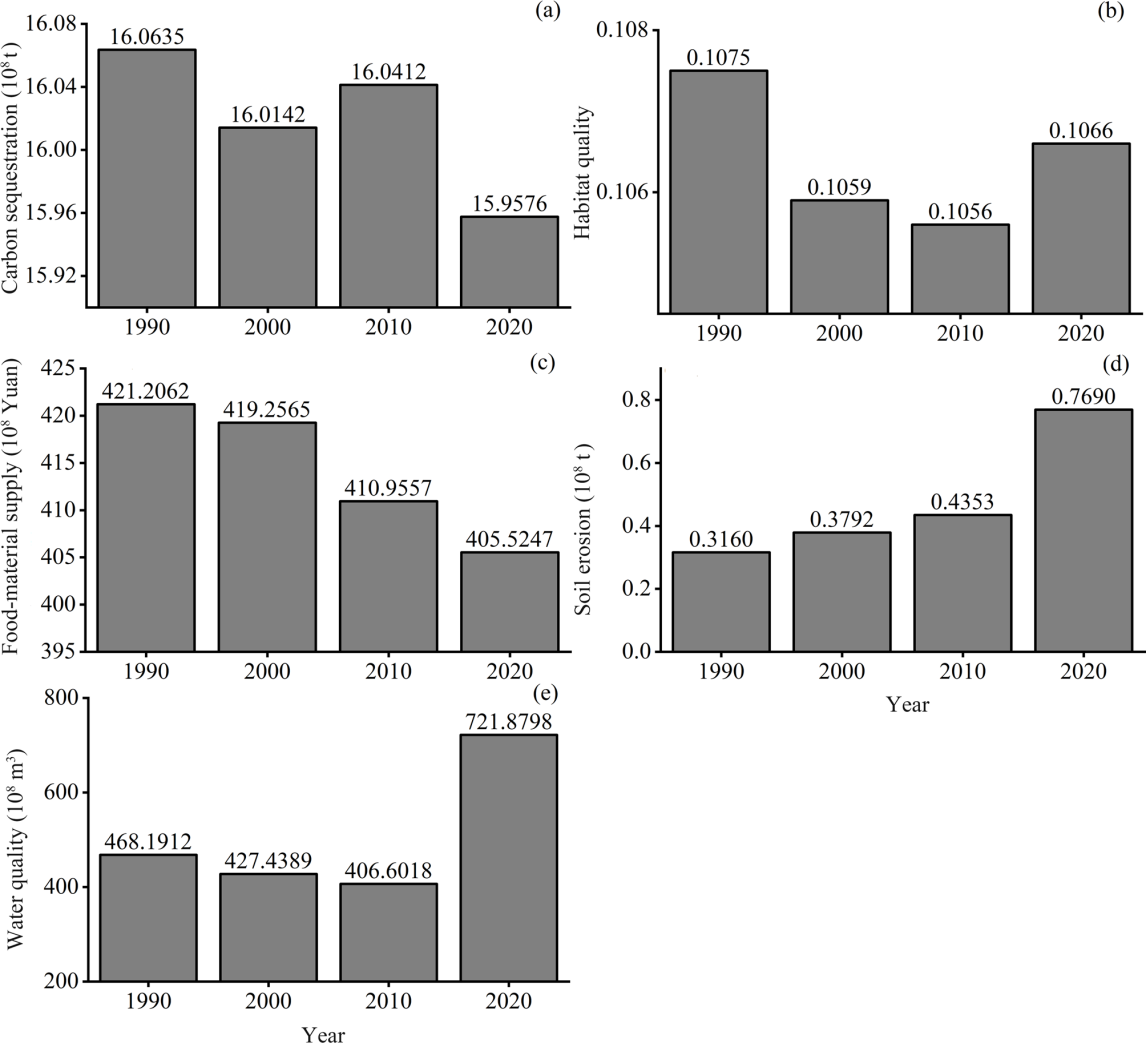
Temporal changes in five ecosystem services in the Huaihe River Eco-Economic Belt during 1990, 2000, 2010, and 2020.

Ecosystem services in the Huaihe River Eco-Economic Belt changed substantially over the past three decades. Carbon sequestration declined from 1.60635 billion tons in 1990 to 1.59576 billion tons in 2020 (Fig. 2a), with the largest reductions in dry land, followed by grassland, and forest (Table 6). Habitat quality decreased from 0.1075 to 0.1066 (Fig. 2b), with the greatest decline in grassland (Table 6). Food-material supply decreased from 42.12 billion Yuan in 1990 to 40.55 billion Yuan in 2020 (Fig. 2c). Dry land, paddy land, and grassland decreased by 1.27, 0.43, and 0.28 billion Yuan, respectively, while water increased by 0.25 billion Yuan due to expansion in water area. Potential soil erosion increased from 31.6 million tons in 1990 to 76.9 million tons in 2020 (Fig. 2d), with significant increases across all land-use types except unused land. Water yield showed a slight decrease from 1990 to 2010 and then a marked increase in 2020 (Fig. 2e). By 2020, all land-use types except unused land exhibited higher water yield than in 1990.

**Table 6:** Changes in ecosystem services of different land-use types in the Huaihe River Eco-Economic Belt.

Ecosystem Service	Year and change	Forest	Grassland	Water	Urban and rural land	Unused land	Paddy land	Dryland
Carbon sequestration (10^8^ t)	1900	12.0598	0.3402	0.2480	0.2933	0.0188	0.7578	1.6862
	2020	11.9858	0.2549	0.3053	0.3933	0.0070	0.7285	1.5981
	Change	−0.0740	−0.0853	0.0573	0.0999	−0.0117	−0.0293	−0.0880
	Change (%)	−1	−25	23	34	−63	−4	−5
Habitat quality	1990	18548.55	6091.1439	2240.899	0	0	0	0
	2020	18867.4	4979.60	2818.27	0	0	0	0
	Change	318.8890	−1111.53	577.373	0	0	0	0
	Change (%)	2	−18	26	0	0	0	0
Food-material supply (10^8^ Yuan)	1990	28.7966	11.2252	10.9920	0	0	110.3017	244.1407
	2020	28.6198	8.4116	13.5310	0	0	106.0418	231.397
	Change	−0.1768	−2.8136	2.5390	0	0	−4.2599	−12.7431
	Change (%)	−1	−25	23	0	0	−4	−5
Potential soil erosion (10^4^ t)	1990	251.194	142.351	0	764.8605	36.1510	112.9683	1394.96
	2020	899.043	395.154	0	1872.404	28.7228	451.0758	2013.20
	Change	647.849	252.803	0	1107.544	−7.4282	338.1075	618.238
	Change (%)	258	178	0	145	−21	299	44
Water yield (10^8^ m^3^)	1990	0.0380	0.0243	0.0020	0.2116	0.0009	0.0470	0.1193
	2020	0.0922	0.0270	0.0098	0.3125	0.0003	0.0896	0.1539
	Change	0.0542	0.0027	0.0078	0.1009	−0.0006	0.0426	0.0347
	Change (%)	142	11	396	48	−64	91	29

**Fig. 3:**
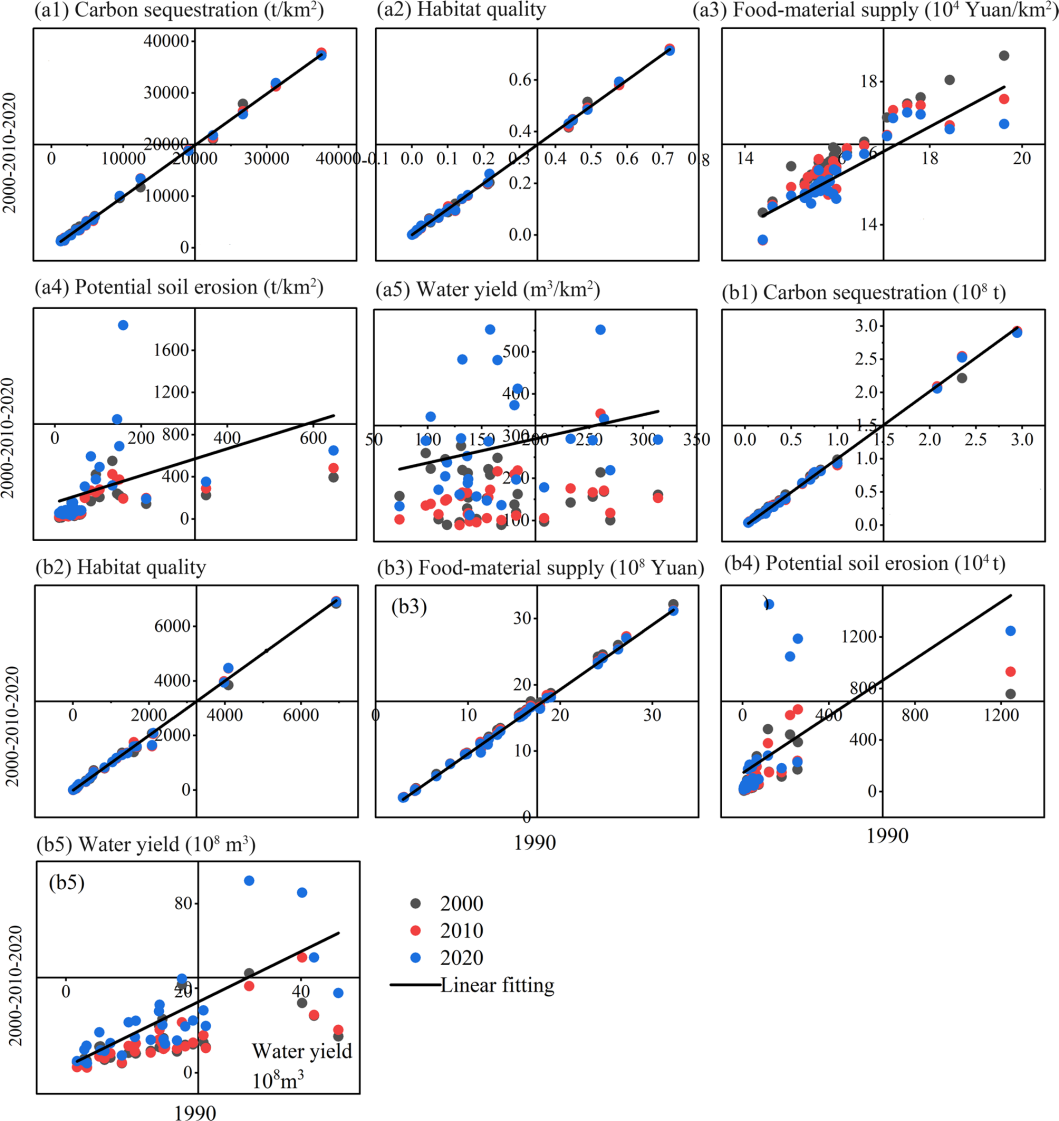
Relationships between ecosystem services from 1990 and 2000, 2010, 2020 at the city level. Panels a1–a5 show ecosystem services per unit area, and panels b1–b5 show total ecosystem services.

**Fig. 4:**
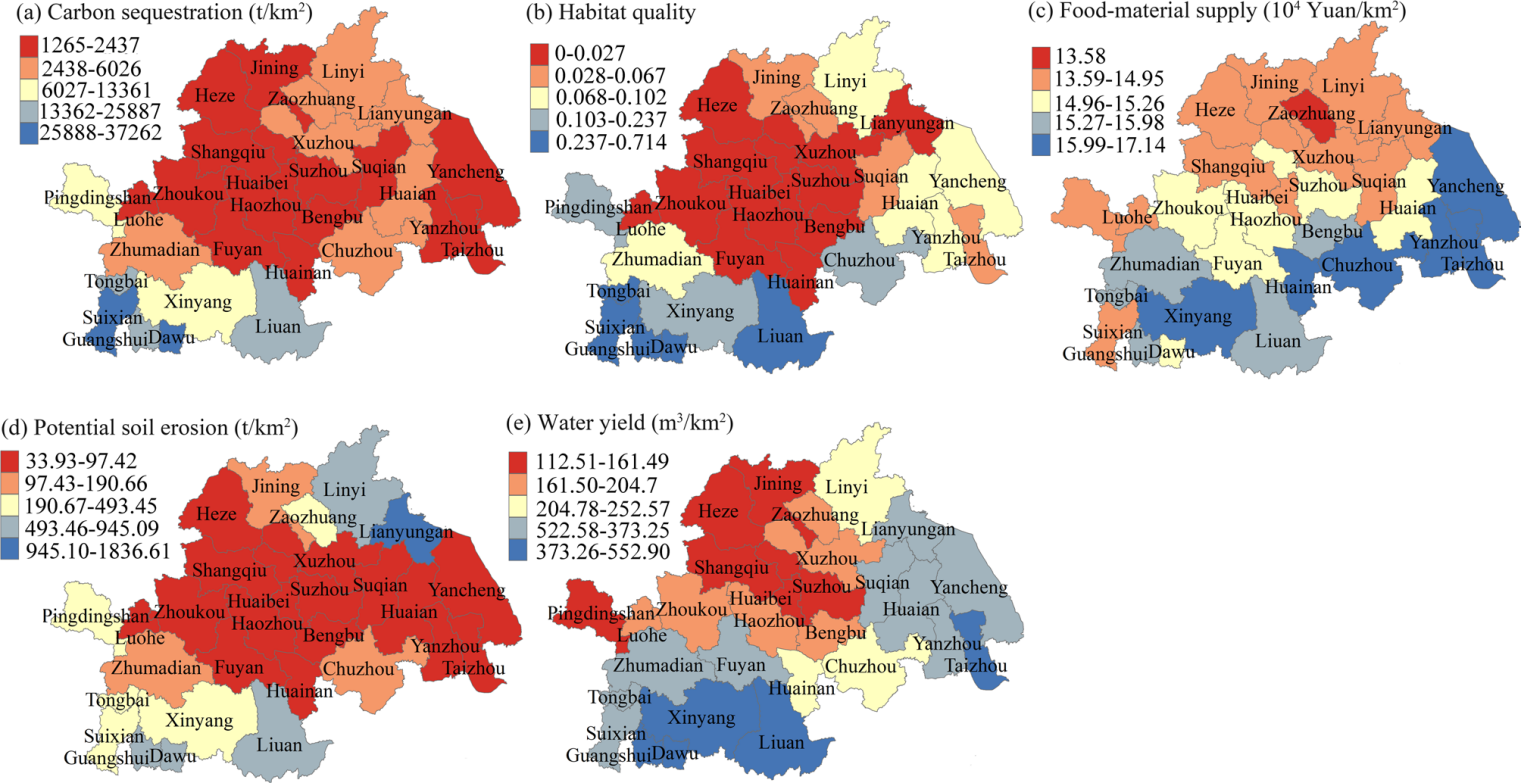
Spatial distribution of ecosystem services per unit area (km²) in the Huaihe River Eco-Economic Belt in 2020.

Consistency in both per unit area and total amount of carbon sequestration and habitat quality across different years was observed at the city-level (Fig. 3). Regions with high carbon sequestration per unit area distributed mainly in the western region (Fig. 4a), and these cities also showed high habitat quality (Fig. 4b). In terms of food-material supply, consistency was observed in the total amount across different years, and a high correlation coefficient was identified per unit area (Fig. 3). Spatially, regions with high food-material supply were mainly located in the southern, while the northern areas had relatively lower values (Fig. 4c). Potential soil erosion remained consistent in both per unit area and total amount across different years, and high-value regions were mainly located in the southwestern region and cities such as Linyi and Lianyungang in the north (Fig. 4d). Water yield also remained consistent in total amount across different years, but per-unit values varied (Fig. 3), with high water yield areas concentrated in southern and coastal regions in 2020 (Fig. 4e).

### Synergies and trade-offs among ecosystem services

**Fig. 5:**
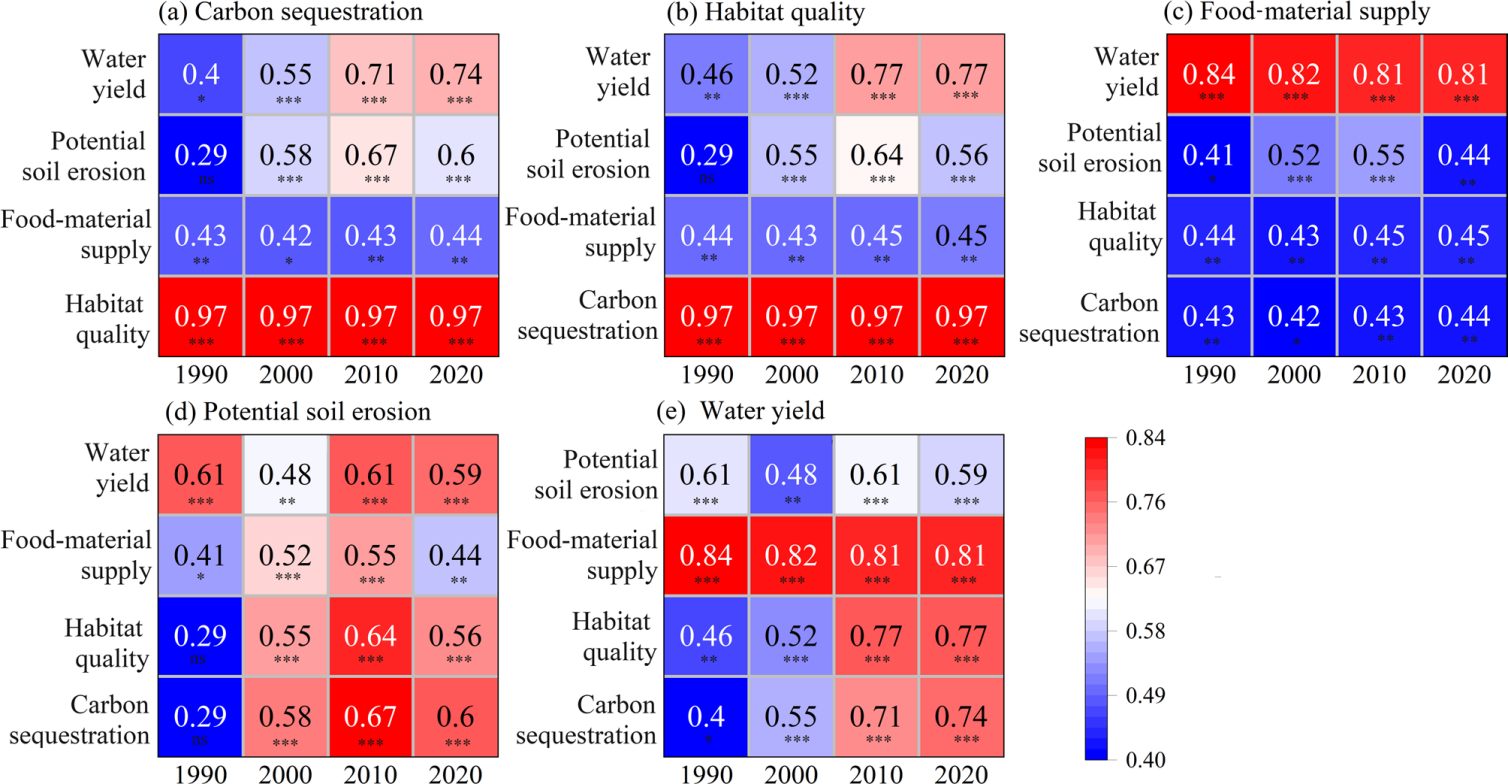
Pearson correlation coefficients among ecosystem services in the Huaihe River Eco-Economic Belt across different years. ^ns^*P* > 0.05, *P < 0.05, **P < 0.01, ***P < 0.001.

Due to limited land-use time points, temporal trade-offs were not analyzed. Spatial correlation analysis across 29 counties and cities indicated positive correlations among all five ecosystem services (Fig. 5). Carbon sequestration and habitat quality exhibited correlation coefficients up to 0.97 (Fig. 5a, b). Food-material supply was positively correlated with other services, mostly with coefficients below 0.50 (Fig. 5c). Potential soil erosion showed no significant correlation with carbon sequestration and habitat quality in 1990, but had significant positive correlations with other services in subsequent years, generally below 0.70 (Fig. 5d). Water yield had substantial positive correlations with all services, especially food-material supply (coefficients > 0.80), and consistently high correlations with carbon sequestration and habitat quality in 2010 and 2020 (coefficients > 0.70) (Fig. 5e).

### Driving factors of ecosystem services

**Fig. 6:**
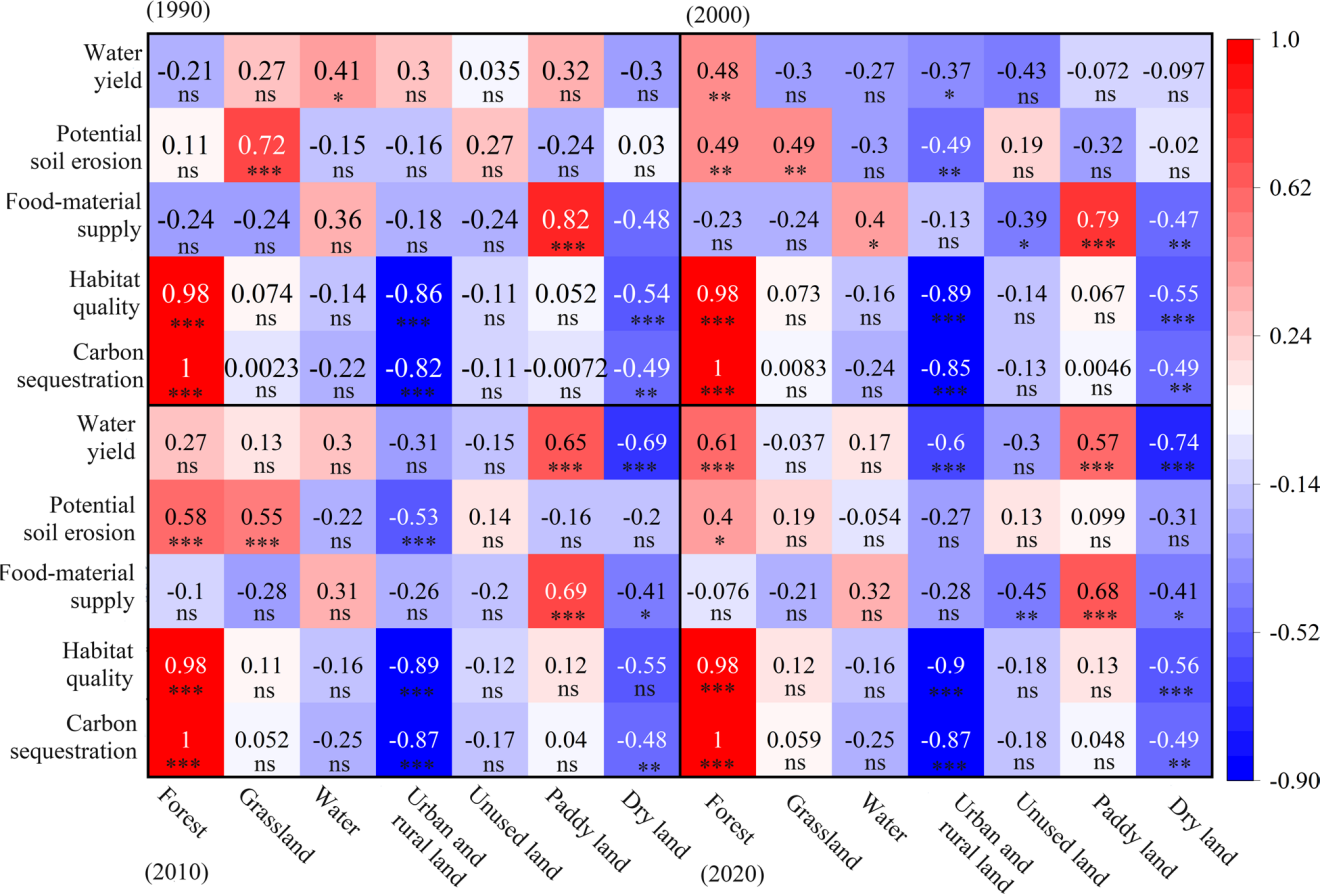
Pearson correlations between ecosystem services per unit area and proportions of seven land-use types in year 1990, 2000, 2010, and 2020. ^ns^*P* > 0.05, *P < 0.05, **P < 0.01, ***P < 0.001.

**Fig. 7:**
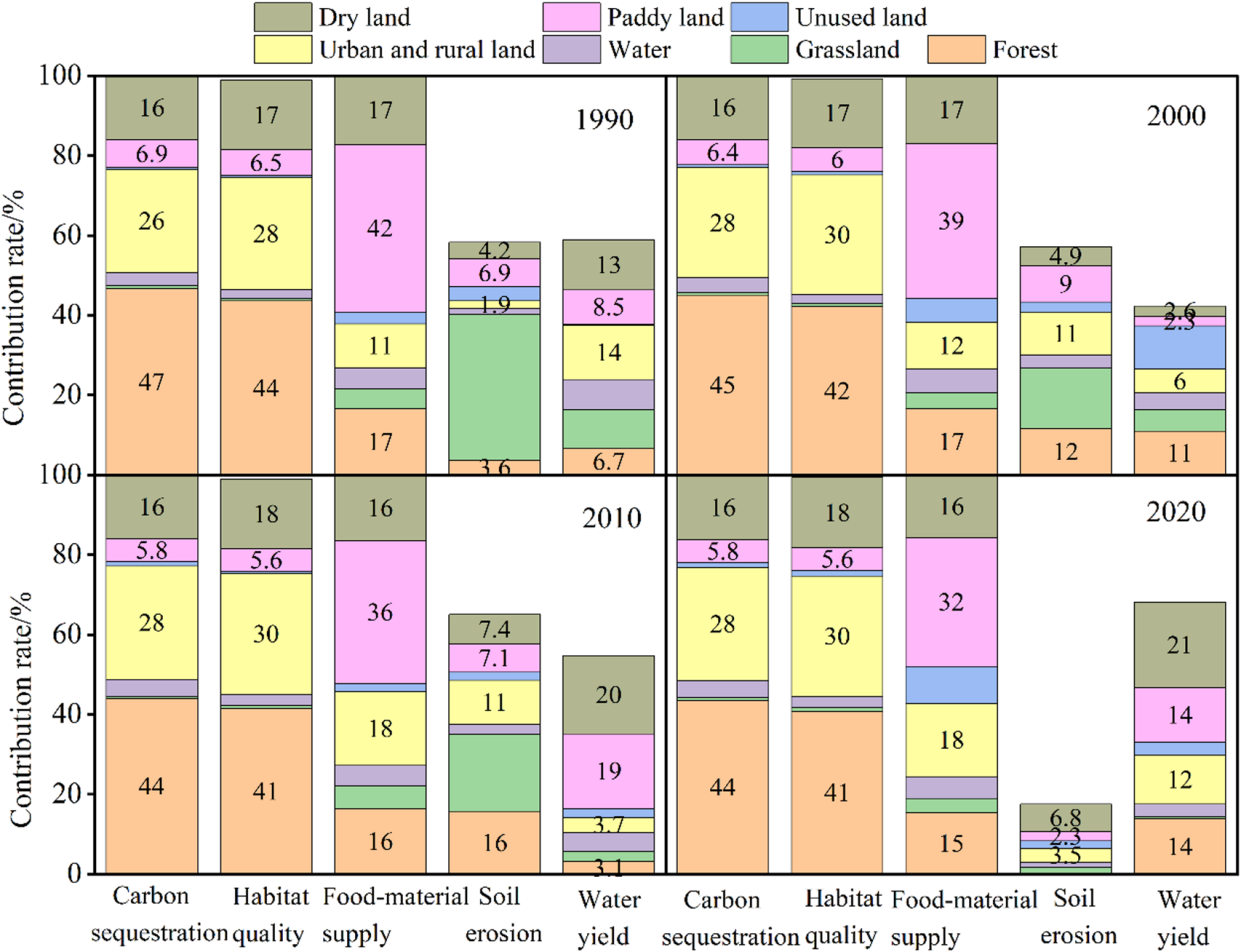
Contribution of land-use proportions to ecosystem services across different years in the Huaihe River Eco-Economic Belt.

The correlation coefficient between carbon sequestration and forest proportion was observed over 0.98. Furthermore, a significant negative correlation was detected with urban-rural land and dry land, especially with urban-rural land, with absolute values exceeded 0.80 (Fig. 6). These findings suggested that the increase in urban-rural land coverage could significantly reduce carbon sequestration in the Huaihe River Eco-Economic Belt. The result of the variation decomposition also given the same conclusion, where forest, urban-rural land, and dry land accounted for 45%, 28%, and 16% of the variation of the carbon sequestration, respectively (Table 7; Fig. 7). The multiple regression indicated that land use proportion could explain 100% of the variation in carbon sequestration (Table 8). Forest, grassland, water, and paddy field could also affect carbon sequestration. Increasing the area of the above land use resulted in a corresponding increase in carbon sequestration (Table 8). The influence of land use on habitat quality was very similar to that on carbon sequestration (Fig. 7). An increase in forest coverage significantly improved habitat quality (explaining 42% of the variation); conversely, an increase in dry land and urban-rural land coverage was associated with a significant reduction in habitat quality (Figs. 6 and 7). Food-material supply demonstrated a positive correlation with water and paddy field proportions, while a negative correlation with the other land use proportions (Fig. 6). Increasing paddy field coverage could significantly improve food-material supply, with a yearly average contribution rate of 37% (Table 7). Moreover, the contribution rates of forest, urban-rural land, and dry land coverage to food-material supply were relatively high (around 15%). As shown from the regression equation, land use proportion could explain 100% of the variation in food production, and increasing paddy field coverage lead to a corresponding increase in the value of food-material supply (Table 8).

Land use proportion accounted for around 50% of the variance in potential soil erosion, with grassland and forest coverage exhibiting relatively high explanatory power of 18% and 10%, respectively (Table 7); while, in 2020, its explanatory power was less than 20% (Fig. 7). The correlation coefficient and regression equation indicated that forest and grassland coverage positively affected potential soil erosion (Fig. 6; Table 8). However, this did not imply that increasing land use above would inevitably result in increased soil erosion. Rather, it is because these areas typically receive higher amounts of rainfall, which can lead to greater erosion. Therefore, for the Huaihe River Eco-Economic Belt, external factors such as changes in precipitation might have a greater impact on potential soil erosion. The dry land, urban-rural land, paddy field, and forest proportions had greater impact on water yield, with explanatory rates of approximately 10% (Fig. 7). Similar to soil erosion, the overall explanatory rate of land use proportion was 56%, and the regression equation revealed that the R^2^ was only 41%, 49% in 2000 and 2010, respectively, indicating that other factors rather than land use, such as changes in precipitation and potential evapotranspiration, may have an important impact on water yield (Table 8).

**Table 7:** Average contribution (%) of land-use proportions to ecosystem services in the Huaihe River Eco-Economic Belt (1990–2020).

Land-use type	Carbon sequestration	Habitat quality	Food-material supply	Potential soil erosion	Water yield
Forest	45	42	16	10	9
Grassland	1	1	5	18	5
Water	4	3	5	2	5
Urban and rural land	28	30	15	7	9
Unused land	1	1	5	3	4
Paddy land	6	6	37	6	11
Dry land	16	17	17	6	14

**Table 8:** Stepwise regression results of land-use proportions and ecosystem service values per unit area across years in the Huaihe River Eco-Economic Belt.

Ecosystem service	Year	Regression equation	Significance
Carbon sequestration	1990	1249.5416 + 512.56 Forest + 24.26 Grassland + 22.63 Water + 29.52 Unused land + 2.03 Paddy land	R^2^=1, *P* < 0.001
	2000	1306.7072 + 511.9379 Forest + 22.8300 Grassland + 22.5647 Water-3.1216 Urban and rural land + 1.7414 Paddy land	R^2^=1, *P* < 0.001
	2010	1246.3166 + 512.3714 Forest + 22.3631 Grassland + 23.2806 Water + 1.8381 Paddy land	R^2^=1, *P* < 0.001
	2020	1242.0711 + 512.6772 Forest + 24.4827 Grassland + 22.5687 Water + 1.8993 Paddy land	R^2^=1, *P* < 0.001
Habitat quality	1990	0.037 + 0.0093 Forest + 0.0029 Grassland + 0.0039 Water-0.0033 Urban and rural land	R^2^=0.99, *P* < 0.01
	2000	0.0389 + 0.0093 Forest + 0.0044 Grassland + 0.0039 Water-0.0032 Urban and rural land-0.0689 Unused land	R^2^=1, *P* < 0.001
	2010	0.0527 + 0.0091 Forest + 0.0032 Grassland + 0.0041 Water-0.0038 Urban and rural land	R^2^=1, *P* < 0.001
	2020	0.03506 + 0.0093Forest + 0.0058 Grassland + 0.0046 Water-0.0026 Urban and rural land-0.1108 Unused land	R^2^=0.99, *P* < 0.001
Food-material supply	1990	18.83-0.0628.83.0628 Forest-0.0765 Grassland-0.0332 Water-0.1882 Urban and rural land-0.1877 Unused land + 0.0301 Paddy land	R^2^=1, *P* < 0.001
	2000	18.8235853-0.0629.8235853.0629 Forest-0.0759 Grassland-0.0331 Water − 0.1883 Urban and rural land-0.1897 Unused land + 0.0301 Paddy land	R^2^=1, *P* < 0.001
	2010	18.82-0.0623.82.0623 Forest.2-0.076.076 Grassland-0.0034 Water-0.0189 Urban and rural land-0.0197 Unused land + 0.0301 Paddy land	R^2^=1, *P* < 0.001
	2020	18.8198 − 0.0629 Forest-0.0762 Grassland-0.0336 Water-0.1879 Urban and rural land-0.1885 Unused land + 0.03028 Paddy land	R^2^=0.99, *P* < 0.001
Potential soil erosion	1990	32.377 + 17.9 Grassland	R^2^=0.52, *P* < 0.01
	2000	81.1073 + 3.4173 Forest + 12.5447 Grassland-1.5956 Paddy land	R^2^=0.42, *P* < 0.01
	2010	51.5022 + 3.9777 Forest + 17.3298 Grassland	R^2^=0.60, *P* < 0.01
	2020	185.618 + 7.965 Forest	R^2^=0.16, *P* = 0.03
Water yield	1990	139.1639 + 4.7499 Grassland + 7.8152 Urban and rural land-1.5570 Dry land	R^2^=0.56, *P* < 0.01
	2000	128.1700 + 1.5946 Forest-126.3546 Unused land + 0.5326 Dry land	R^2^=0.41, *P* < 0.01
	2010	109.1850 + 0.6949 Forest + 1.6571 Paddy land	R^2^=0.49, *P* < 0.0001
	2020	167.5381 + 3.8004 Forest + 3.3581 Paddy land	R^2^=0.67, *P* < 0.01

## Discussion

Carbon sequestration, habitat quality, and food-material supply declined in the Huaihe Eco-economic Belt, mainly due to land-use change (Table 9). Between 1990 and 2010, decreases of 6,424 km² and 2,950 km² in dry land and grassland, respectively, substantially reduced carbon storage. Although forest area decreased by only 109 km², its high per-unit carbon density contributed meaningfully to the decline. Habitat quality decreased primarily due to grassland loss, while increases in water bodies partially compensated for habitat degradation. The habitat quality increased markedly during 2001–2018 in the Yangtze River Basin, which can be attributed to the increase of wetland and forest^38^. The different trend between the two major basins may rise from the distinct land use type, the Yangtze River Basin is dominated by grassland, while farmland cover the most area of the Huaihe Eco-economic Belt (Fig. 1). Food-material supply decreased as extensive conversion of paddy land and dry land to urban and rural land reduced production; declines in grassland and forest posed additional risks to raw-material supply. Although expanded water bodies increased food-material supply, the gains were relatively small compared with losses. Dang et al. (2025) found that the food-material supply in the Yellow River Basin increased 88% during 1990 to 2020, and this pattern was mainly due to the land use change while the social-economic factors such as population and GDP are of low importance^39^. Potential soil erosion and water yield generally increased; land-use captured part of the variance, changes in precipitation and erosivity likely played important roles. For instance, precipitation increased from 990 mm in 1990 to 1,107 mm in 2020, coinciding with increased water yield from 46.8 to 72.2 billion m³.

Spatially, ecosystem services exhibited positive synergies: areas with high values of one service tended to have high values of others. Synergies were strongest between carbon sequestration and habitat quality (*r* > 0.90) and between water yield and food production (*r* > 0.80). Combined evidence from correlations, hierarchical partitioning, and regression indicates that spatial variation in carbon sequestration, habitat quality, and food-material supply is predominantly explained by land-use proportions: forests increased carbon storage and habitat quality, while urban and rural land and dry land reduced them. Potential soil erosion and water yield were also influenced by precipitation, especially in high-precipitation regions.

Though we obtained important insight of the ecosystem services values and dominant land-use type controlling them. Limitations still exist, uncertainty in InVEST inputs (e.g., carbon densities sourced from literature), which may introduce bias. Data were from four years, reducing the ability to capture long-term dynamics. Future work should prioritize improving the reliability and continuity of input data and incorporate scenario analysis and uncertainty quantification. In addition, we did not concern the interactive effects of socio-economic drivers, policy changes, or climate variability in a quantitative way, further work will integrate these factors and use structural equation model to obtain more comprehensive understandings. Furthermore, we did not consider the nonlinear effects of land-use type on regression results, machine learning such as XGBoost would be a useful method to deal with this issue in the future work.

**Table 9:** Land-use transfer matrix between 1990 and 2020 in the Huaihe River Eco-Economic Belt (km²).

	Forest	Grassland	Water	Urban and rural land	Unused land	Paddy land	Dry land
Forest	15,572	1706	470	690	14	2180	3027
Grassland	2062	3403	766	633	34	562	3555
Water	409	339	6448	1080	7	1917	2654
Urban and rural land	306	264	1410	7349	14	4823	16,376
Unused land	17	33	447	34	20	75	115
Paddy land	1966	468	2377	7998	10	33,558	5178
Dry land	3218	1852	3051	22,450	66	6692	93,709
1990 sum	23,659	11,015	12,854	30,542	741	51,555	131,038
2020 sum	23,550	8065	14,969	40,234	165	49,807	124,614
Absolute change rate	−109	−2950	2115	9692	−576	−1748	−6424
change rate (%)	−0.46	−26.78	16.45	31.73	−77.73	−3.39	−4.90

## Conclusion

This study employed the InVEST model to investigate the dynamics of soil carbon storage, habitat quality, food-material supply, potential soil erosion, and water yield in the Huaihe River Eco-Economic Belt at 10-year intervals between 1990 and 2020. By analyzing land-use proportions, the driving factors behind these changes were identified. The major findings are as follows:

(1) Soil carbon sequestration declined from 1.60625 billion tons in 1990 to 1.59575 billion tons in 2020, primarily due to reductions in carbon storage in dry land, grassland, and forest. Habitat quality decreased, largely driven by grassland loss. Food‑material supply also declined, from 42.1 billion Yuan in 1990 to 40.6 billion Yuan in 2020, mainly due to decreases in dry land, paddy fields, and grassland. Potential soil loss increased continuously, while water yield first decreased and then rose, reaching 72.1 billion m³ in 2020. These changes were strongly influenced by increased precipitation.

(2) Ecosystem services exhibited spatial synergies, with high values of one service often coinciding with high values of others. Habitat quality and carbon sequestration showed a strong correlation, while water yield and food‑material supply were also highly correlated.

(3) Forests, urban and rural land, and dry land were the most influential land‑use types affecting carbon sequestration and habitat quality, explaining approximately 43%, 29%, and 16% of the variation, respectively. Food‑material supply was strongly influenced by paddy field. Forest and grassland proportions affected potential soil loss, while dry land, paddy fields, urban and rural land, and forests were key drivers of water yield. In addition to land‑use change, climatic factors such as precipitation played critical roles in shaping soil erosion and water yield.

Overall, this study provides important insights into the evolution of ecosystem services in the Huaihe River Eco‑Economic Belt over the past three decades and identifies the primary land‑use drivers of these changes. The findings highlight the importance of sustainable land‑use practices that prioritize conservation and restoration of natural ecosystems, particularly forests and grasslands, to enhance carbon storage, habitat quality, and food production. Future research should focus on improving the reliability and continuity of data to deepen understanding of long‑term ecosystem service dynamics and to inform effective policy interventions that support sustainable development in this critical economic region.

## Data Availability

The datasets used in the current study available from the corresponding author on reasonable request.
